# The complexities of athlete development in North American professional sport

**DOI:** 10.3389/fpsyg.2026.1804236

**Published:** 2026-05-18

**Authors:** Dale Lablans, Kathryn Johnston, Joseph Baker

**Affiliations:** 1 School of Kinesiology and Health Science, York University, Toronto, ON, Canada; 2 Faculty of Kinesiology and Physical Education, Tanenbaum Institute for Science in Sport, University of Toronto, Toronto, ON, Canada

**Keywords:** athlete support, athlete transitions, career progression, elite athletes, high performance sport

## Abstract

As the number of roles and positions within professional sport organizations dedicated to athlete development grows, coaches and other interest-holders continue to look to empirical evidence to guide their approaches and inform decision-making. Unfortunately, evidence to date is limited, leaving practitioners wondering how they can best support and make positive change in the athletes under their care, especially when these athletes are typically adults and at the most elite stages of their sporting careers. Using a narrative review approach, this work draws from areas within sport science including talent development, research methods, and athlete support to highlight some of the contemporary issues faced by practitioners in these roles. The main challenges identified include (a) definitional ambiguity, (b) measurement and analysis challenges, (c) restrictive theoretical modeling, and (d) implementation science barriers. Overall, based on the limited, high-quality, empirical evidence to date, decisions on what is “best” for professional athletes at this stage of their careers remains elusive. Caution should be taken when making assumptions, and applying a “one size fits all approach” for athlete development in early adulthood.

## Introduction

The Anaheim Ducks of the National Hockey League (NHL) made an announcement that puzzled many in the hockey community in 2023. They revealed that their new prized draft pick, Leo Carlsson, the Swedish-born third overall pick in the 2023 NHL draft, would have a game limit imposed for the upcoming 2023–2024 NHL season. Given Carlsson's potential to be a future star of the organization, combined with his ability to add immediate value to the young team, this decision to predetermine the number of games he will play raised questions. It could be that adopting this type of imposed limit strategy would help him prioritize his development as an athlete, a strategy that is not very common in the NHL. Since this was an atypical approach, and given a player's traditional performance statistics are based on yearly/season-long averages, this had the potential to impact Carlsson's future contracts, the team's potential wins and losses, and the franchise's short-term revenue. However, it is likely the organization believes this development-focused approach will help unlock Carlsson's “potential” as a hockey player for the long term.

This unconventional strategy by the Ducks exemplifies the complexities and evolving landscape involved in elite and professional athlete development. That said, while this approach appears rooted in developmental priorities, what is left to be determined is whether or not this approach actually supports Carlsson's development. Does a game restriction mean Carlsson will have greater opportunities to rest and recover, and/or to work with staff members who are implementing a multidisciplinary approach to athlete development? This will be hard to decipher, but either way, it marks an important change in process and protocol signaling a shift in thinking about an athlete's journey in professional sport.

At the center of decisions such as these are staff members who are increasingly being hired into “athlete development” roles. Within the major North American professional sports leagues (NAPS)—the National Football League (NFL), National Basketball Association (NBA), Major League Baseball (MLB), Major League Soccer (MLS), NHL, National Women's Soccer League (NWSL), Northern Super League (NSL), Professional Women's Hockey League (PWHL), and Women's National Basketball Association (WNBA)—roles dedicated to athlete/talent development are growing at a rapid rate [for some current news articles highlighting this notion, see (Global Sports Desk, 2025; Bondy, 2025)]. It is hard to ignore, however, that decisions in professional sports are heavily influenced by financial incentives. Often these incentives are tied to short term success (i.e., measured through immediate outcomes like wins, rankings and championships), which can come at the expense of prioritizing longer term development for athletes. With staff and player contracts being relatively short, coaches under pressure to win may overplay early-maturing athletes because they provide immediate competitive advantages. This raises the question: *can long-term athlete development and wellbeing coexist in a professional sport setting*?

Human performance is neither simple nor linear. Scholars across multiple domains, including sport, music, education, business, and the arts, have long sought to understand what differentiates expert performers from their peers. Early work in this field was largely framed through the “nature vs. nurture” debate, attributing elite performance primarily to innate ability or environmental influences (Klissouras, 2001; Singh, 2012). More recent scholarship, however, has adopted multifactorial and dynamic perspectives, recognizing expertise as the product of interacting individual, social, and environmental factors with increasing attention to the role of the ecosystem in which they are embedded (Baker et al., 2003; Baker and Horton, 2004; Phillips et al., 2010; Davids and Baker, 2007). Championships in professional sports are won by teams, not isolated athletes. Consequently, growing interest has emerged in understanding the characteristics of teams that perform effectively. While it is commonly assumed that teams that function well together (often termed “team effectiveness”) achieve better outcomes, this relationship is neither automatic nor guaranteed.

Importantly, team effectiveness should not be considered solely as an outcome (e.g., wins or rankings), but as a developmental construct, reflecting how well a team coordinates its efforts, adapts under pressure, and maintains collective functioning over time (Peralta et al., 2018). Contemporary perspectives define team effectiveness as both the quality of collective performance and the team's long-term viability (Carron et al., 2009). Within sport, this includes components such as task and social cohesion, communication quality, role clarity, shared mental models, and leadership under pressure (Collins and Durand-Bush, 2015). These elements are not static; instead, they evolve through interaction, experience, and deliberate development. To investigate these interactions, (Stewart et al. 2024b) performed a review to investigate the important individual, team, and external inputs along with mediators for/of team effectiveness. The authors noted that based on the frameworks identified in their review, athletes need to possess not only the requisite disciplinary knowledge and skillset, but also demonstrate open mindedness, adaptability, pragmatism, humility, honesty, empathy, respect, consistency, and the ability and capacity to take responsibility for team effectiveness (Stewart et al., 2024a; Alfano and Collins, 2021; Stewart et al., 2024b). For performance support teams to be effective, there must be enough human capacity to deliver the desired outcome (i.e., number, size and diversity of team matters); however, as capacity increases, so does increased potential for complexity, conflict, and unintended ineffectiveness (Stewart et al., 2024a). Importantly, clarity regarding role understanding and effective communication can serve as a potential antidote to such negative consequences (Stewart et al., 2024a; Reid et al., 2004). With regards to mediators, team culture and the underpinning trust required to facilitate a psychologically safe environment appear to be important (McEwan and Beauchamp, 2014). Overall, the authors reported that team effectiveness depends on more than simply assembling diverse experts; individual, team, and environmental inputs interact with team processes and emergent states, and key factors such as expertise, leadership, team composition, and organizational context enable these inputs to translate into effective performance outcomes.

To complement the perspectives of those in roles that support athlete/player development, (Till et al. 2021) examined 136 male youth academy soccer players' perceptions of their talent development environments (TDEs) and compared these perceptions across the English academy categorization system. These players were classified as being part of the Professional Development Phase, which is traditionally the stage of development prior to being offered a professional contract, and their findings illuminated that players from higher-category (CAT1) academies reported more positive perceptions of support networks and holistic preparation than those from lower categories, with holistic preparation rated lowest in CAT2–CAT3 academies. The authors noted that staff, including coaches, should therefore seek to support players' personal and social excellence, as well as physical, technical, and tactical aspects. The findings suggest strong support networks and teams that adopt more holistic development practices observe enhanced player experience and club progression relative to their lower tier counterparts (Till et al., 2021).

While youth and adolescent development pathways have received extensive empirical attention, comparatively little is known about development processes at the professional level, especially from a holistic perspective. This gap is consequential, as professional athletes operate within distinct performance, contractual, financial, and organizational constraints that likely shape their developmental experiences in ways that differ from earlier stages. Accordingly, this paper seeks to scan, retrieve, and synthesize literature addressing the significant challenges associated with conducting empirical research on NAPS athlete development. In doing so, the paper also aims to illuminate evidence-informed strategies to minimize these challenges, thereby supporting researchers and practitioners in advancing knowledge and practice in professional athlete development. Professional sports leagues differ globally and the North American system was chosen for this investigation due to the unique social and cultural constraints within this context. For instance, players typically (a) spend time moving between different systems across their development (e.g., hockey players might move between youth, junior, and college systems before reaching the professional level), (b) enter the professional level after being “drafted” by a team, and (c) generally travel large distances across a season due to the unique geography of playing in the United States and Canada (with the exception of the NFL, which does not usually play games outside the continental United States). Unfortunately, most of the research conducted on the NAPS has focused on men's sports. While it was the intention to present evidence from both genders, this review primarily represents research conducted on men's sports.

## Methods

To address the research question, a narrative review was chosen to help scan and synthesize research in the area of athlete development for professional athletes. In this review, (Baker et al. 2023) definition of athlete development was used, which describes it as a process involving growth or change, closely tied to learning, and influenced by the environment. Narrative reviews enable a broader exploration of related fields (e.g., spanning multiple domains beyond sport) and its accepted subjectivity provides an opportunity for a comprehensive evaluation in spite of the minimal available empirical evidence on the real-world (multi-faceted) aspects of athlete development (Greenhalgh et al., 2016). Further, exploring NAPS athlete development through this lens enables this paper to be situated within the broader domain of athlete development and for stories, anecdotal evidence, and sources outside the peer-reviewed landscape to be incorporated into the paper's discussion.

### Approach

To conduct the narrative review, Google Scholar, PubMed, and Research Rabbit were utilized as the main search engines and databases. To determine search terms used in these databases, a snowball approach was used to gather articles on professional athletes, and on athlete development theories and practices more generally. To do this, reference lists were scanned and articles mentioning either professional athlete, or athlete development in their titles were compiled and read. Of these articles, recurring terms were identified, which expanded the search terms to include: “talent development,” “expertise,” “athlete development models,” “national sport organizations,” “developing athlete,” “masters athlete,” “elite athlete,” “professional athlete development,” “long term athlete development,” “foundation, talent, elite, mastery,” “athletic skill model,” “constraint led approach,” “dynamical systems theory,” “sport and national pride,” “masters athlete participation,” “youth sport participation,” “sport and abuse,” “psychological safety in sport,” “sport and national pride,” “professional sport and economic benefit,” “narrative review,” “narrative review methodology,” “sport participation and positive development,” “negative impact of sport,” “athletes' as role models,” “inclusivity in sport,” “professional sports and entry draft,” “measurement in sport,” “research communication in sport,” “research methods in sport.”

Once databases were searched using the phrases and terms listed above, articles were identified based on relevance to the subject. Selected papers were read in full, once for general understanding and a second time at which point DL summarized the contents and highlighted pertinent information. Throughout the process of data collection, DL conducted information processing through a continuous recursive process of information synthesis and subsequent reflective note taking to develop understanding. To formulate the results section of this narrative review, themes were created. This process involved DL reading and re-reading the identified articles and assigning labels to the articles based on their sample (i.e., professional sport or otherwise), their focus/foci (e.g., relationship to athlete development or developmental environments, measurement and methodological approaches etc.), the article's finding(s), and the limitations raised by the authors of the articles to help illuminate the methodological and practical challenges that might have been faced. The labels were then used to create clusters into common themes. These themes were arranged into four main “challenges” to help address our research question. Importantly, while there was an opportunity to create more than four themes, the research team felt like many of themes could be merged together (i.e., “Limitations of Applying Findings” and “Building Sport Science Bridges” were initial themes, that were merged into “Implementation Science Barriers”), to make an approachable and less-repetitive set of challenges for researchers and practitioners to consider.

**Figure 1:**
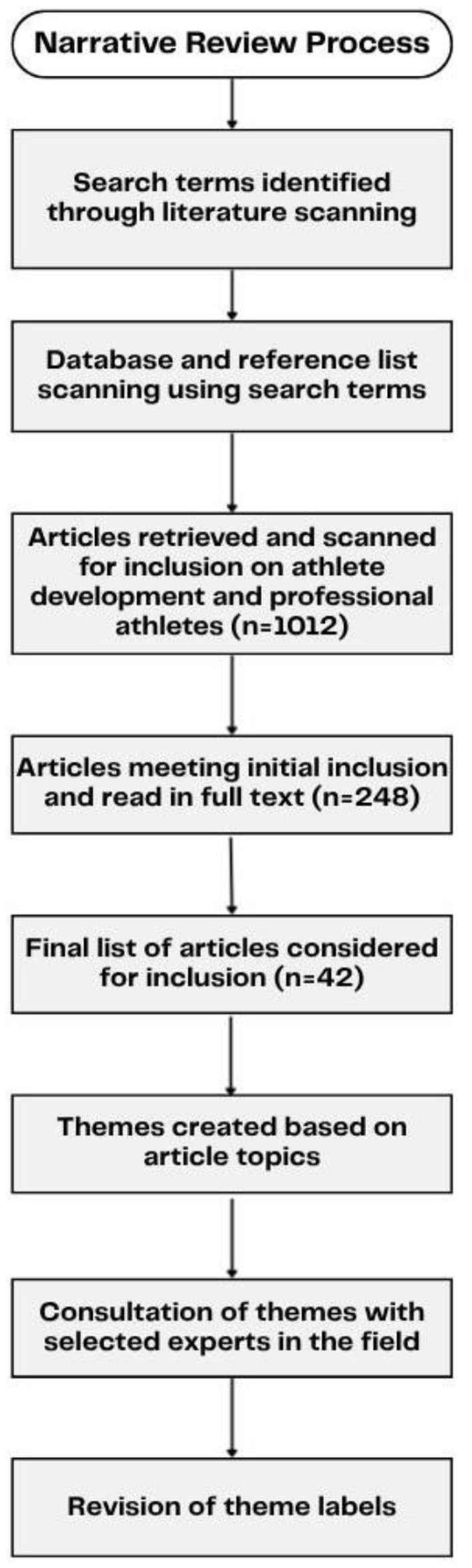
Flow diagram of narrative review process.

The identified challenges were then revisited and confirmed in consultation with practitioners and colleagues in the field. To do this, DL consulted with development coaches on several professional teams to confirm the four broad challenges from a practical standpoint. The developmental coaches in the field were hand selected by DL (i.e., a convenience sample), and a snowball sampling approach was used to gain more perspectives (i.e., DL asking a coach, which other coaches would be beneficial to speak with). Comments and feedback were compiled, all of which were minor suggestions for areas to expand research searches within, of which DL performed searches for more readings in the area, further bolstering the evidence within the identified challenges. Importantly, the feedback from development coaches did not result in the adding or subtracting of a challenge (please see Figure 1 for a flowchart of the process used for the present narrative review).

## Results

Through the narrative review process, four categories of information were identified that capture some of the most pressing challenges with respect to athlete development facing researchers and practitioners in the professional sport landscape in North America.

### Challenge 1—definitional ambiguity

Definitional ambiguity related to the term “athlete development,” and neighboring terms like “talent development,” and “player development,” results in significant barriers to understanding this area of research and practice. The word “development” itself is quite blurry, having multiple definitions that vary in context and priorities. For instance, with a medical and health lens, (Cameron and Schell 2021, p. 1), share that “Human growth and development are characterized by the way in which we change in size shape and matrix relative to the passage of time. Similarly, (Kagan 1991, p. 4) described the study of human development as “describing patterns of constancy and change across the lifespan and identifying the underlying processes that account for these patterns.” with “development” being a change that occurs over time and that has a direction. Within the sport context, the term “athlete development” is frequently used when describing the journey from early participation to high performance structured competition; however, it is a term that is rarely defined. It is more common to see papers address the desired outcomes from successful development in sport and through sport [see (Côté and Erickson, 2016) as an example]. Generally, however, in the sport science literature, athlete development is understood as a longitudinal and multifactorial process through which individuals acquire sport-specific skills, psychological competencies, and adaptive capacities across different phases of their sporting career (e.g., Côté and Fraser-Thomas, 2007; Côté, 1999; Balyi and Hamilton, 2004).

Adding another layer of complexity, the term “professional sport” is often intertwined with “elite,” “expert,” “high performance,” and “world class” sport (McAuley et al., 2022). Although these terms are frequently used interchangeably, they are not synonymous. “Professional” typically denotes contractual and financial status, whereas “elite” or “world-class” often refer to performance standard or competitive ranking. This conceptual ambiguity complicates efforts to define the population under investigation and may contribute to inconsistencies in how professional athlete development is operationalized across studies. Without clear definitional criteria, it becomes difficult to determine whether studies are examining occupational status, performance caliber, or expertise attainment, each of which may reflect distinct developmental processes. This blurry terminology (i.e., words that are unclear or poorly defined) along with how multi-faceted development—specifically athlete development—is one of the main limitations of rigorous research and knowledge translation. In contrast to disciplines like physics and biology, which rely on shared terminology to articulate phenomena, the literature on athlete development lacks a standardized vocabulary, with many terms (i.e., athlete, player, talent, etc.) being used interchangeably. (Johnston et al. 2023) argued for a balance between accepting conceptual vagueness and delineating sharp boundaries to quantify certain terms or phenomena. Determining which terms require greater clarity may be difficult, but any term involving measurement (like athlete development) requires precision (Johnston et al., 2023).

Ultimately, achieving consensus on terminology and definitions is crucial for advancing the field of athlete development and ensuring the efficacy of athlete development efforts within professional sports organizations. Conceptual clarity is not limited to semantics involving concepts and their definitions, but rather influences all aspects of the research process, from measurement and the statistical methods used to answer a research question to interpreting what the evidence tells us and how it may be best applied (Bringmann et al., 2022). Without a shared understanding of core concepts and practices, many elements of athlete development remain elusive, risking misdirection of resources and hindering organizational growth and success.

To help gain conceptual clarity surrounding athlete development, (Baker et al. 2023) advocated for reframing the term without reliance on the notion of talent. The term talent is itself blurry (Johnston et al., 2023; Baker and Wattie, 2018; Grainger et al., 2024; Johnston and Baker, 2022), pre-loaded with biases about what is trainable/can be developed through rigorous practice, which makes its value in athlete development contexts questionable. For this reason, using “athlete development” may be more beneficial for conceptual clarity reasons, than “talent development.”

Similarly, there is conceptual blurriness surrounding where professional athletes fit in the taxonomy of competition levels, which may influence the degree to which data and results are connected to this population. While some studies consider professional athletes “elite” (i.e., Jordet and Elferink-Gemser, 2012), others are not so quick to merge elite populations with professional athletes (Swann et al., 2015). To date, a substantial body of work has identified the characteristics that distinguish expert from novice performers, which helps explain mechanisms of expertise but offers limited insight into how performance is sustained during the expert stage itself. Understanding what influences the duration of peak performance is particularly important, as traditional models assume inevitable decline with age. This can present a challenge for researchers to draw conclusions about research findings with professional athletes, as sample demographics may not always be clearly stated for comparisons to be made.

### Challenge 2—measurement and analysis challenges

Relatedly, the second challenge captures the difficulty that researchers and practitioners have in measuring “development” in meaningful ways. In a complex environment like sport, many measurement challenges exist for researchers and practitioners. Perhaps most significant are the considerations related to sampling the population. Not dissimilar to other exceptional populations of study (i.e., including expert performers in music, mathematics, chess; Ericsson and Charness, 1994), elite athletes are rare by definition. Consequently, research with elite athlete populations often involves small samples for various reasons, including the small size of target groups [e.g., there are only 736 active roster NHL players and 138 active roster Professional Women's Hockey League (PWL) players], difficulties in accessing protected groups such as athletes within professional organizations, prohibitive data collection costs, or high dropout rates among study participants (Kiefer and Martin, 2022). Additionally, there may be limited diversity and representation in some sports, which have implications for sampling. In sports that are more homogenous in terms of who participates [e.g., 93% of all NHL players in the early 2020s identified as Caucasian (Whyno, n.d.)], inference to under-represented groups may be limited. Women in sport are also recognized as an under-represented group when it comes to sport research (Johnston et al., 2018; Baker et al., 2020). We are only recently seeing expansion of professional leagues in North America with the newly formed professional women's hockey league (PWHL), soccer leagues (NWSL; NSL), the WNBA (new in terms of the expansion to Canada), and the Women's Pro Baseball League (currently under development). Given how relatively new these leagues are compared to men's equivalent leagues, the field is lagging for research on women's performance and development, highlighting an area ripe for investigation.

Historically, most studies within this domain have relied on retrospective recall or cross-sectional methods, which either look at athlete development through a rearview mirror (i.e., what worked in the past for currently successful elite athletes) or provide snapshots of athlete development at particular points in time (Baker et al., 2019; Harry et al., 2024). While these approaches have their merits (e.g., being less time-consuming and less resource-intensive), they have significant limitations. For instance, retrospective recall is often subject to memory bias (Müggenburg, 2021), and cross-sectional designs, while useful for identifying relationships, are incapable of establishing causality (Wang and Cheng, 2020) or tracking developmental trajectories over time. Recognizing these limitations, researchers have increasingly advocated for a shift toward longitudinal research in elite sports, which offers unique advantages over other approaches. Longitudinal studies have the potential to establish cause-and-effect relationships, offering a more nuanced understanding of how specific training practices, environmental factors, or psychological attributes influence long-term athlete development (Cobley et al., 2014). Moreover, longitudinal research can track the stability, or variation, in key developmental variables (e.g., physical performance metrics, mental resilience, or skill acquisition) over time, providing a richer context for interpreting changes in athlete performance. Importantly, longitudinal research also allows for the tracking and monitoring of athletes who are unsuccessful in the athlete development pathway, which is critical for overcoming the “survivorship bias” inherent in much of the work in this area (Baker et al., 2022).

However, executing longitudinal research within elite athlete development is difficult. Access to elite athlete populations is notoriously hard to obtain due to the protective nature of sports organizations and the high demands placed on athletes. Organizations can be reluctant to allocate the necessary resources, such as time, funding, and access to athletes, for conducting thorough longitudinal studies. Additionally, the high turnover rates among both athletes and staff in elite sport systems can lead to significant attrition in longitudinal studies, resulting in incomplete data sets and reduced statistical power. The dynamic nature of elite sports (including professional sports), characterized by rapidly shifting organizational goals, priorities, and coaching philosophies, further complicates longitudinal research efforts. These changes can disrupt research continuity, alter the availability of resources, and necessitate frequent adjustments to study protocols, all of which can undermine the consistency and reliability of data collection.

Professional athletes' demanding training and competition schedules present another layer of complexity. These schedules often involve frequent travel and exposure to highly variable environments, such as different climates, altitudes, and time zones, all of which can introduce unaccounted-for noise into the data, complicating how research results are interpreted and applied (Baker et al., 2023). The inconsistency in scheduling and the challenges of maintaining regular data collection amidst these variables can lead to data that is difficult to compare across different time points.

In addition to these logistical challenges, researchers must navigate the organizational policies and regulations that govern data collection in professional sports. For example, in the NHL, the Players Association imposes strict guidelines on what types of data can be collected and under what conditions. Certain metrics cannot be collected during games, and data collection outside of official work hours is often prohibited. Furthermore, there are protocols for the deletion of individual athlete data when athletes leave a team, whether due to retirement, trade, or injury. Testing periods are also tightly controlled by league-approved protocols, limiting the flexibility of researchers to design and implement their studies. These restrictions pose significant hurdles for researchers, requiring them to be innovative and adaptive in their methodological approaches. One avenue forward may be to consider qualitative and mixed-method data collection processes which could help develop this area. Tønnessen et al. (2025) used an intrinsic case study design to capture in-depth information on the development process of a world-class soccer player. This approach was purposely chosen to provide a comprehensive and balanced exploration of the athlete's developmental journey by integrating both objective (i.e., training data) and subjective (i.e., interviews) perspectives. In elite and professional sport, where sample sizes are small and access is limited, rich case studies can provide insights that large-scale quantitative studies cannot realistically obtain.

### Challenge 3—restrictive theoretical modeling

As academic interest in human excellence and achievement has evolved, the study of athlete development has morphed into a field of research of its own (Till and Baker, 2020), leading to a proliferation in athlete development models (ADMs). Early work by (Bloom 1985) classified development into three stages (i.e., initiation, development, and perfection). While this three-stage model has been adapted and refined, the notion that development is discrete underpins many of the models currently in use. For example, the Long Term Athlete Development (LTAD) model indicates athletes progress through six stages in their high-performance career: FUNdamentals, Learning to Train, Training to Train, Training to Compete, Training to Win, and Retirement (Balyi and Hamilton, 2004). Similarly, the FTEM model (Foundation, Talent, Elite, and Mastery; Gulbin J. P. et al., 2013) includes four stages with the final stage of the model capturing an athlete's sustained success over multiple “cycles.” The FTEM model focuses on development in Olympic-level athletes, and as a result its relevance for those in professional team sports is still to be determined. It is not clear where and if professional athletes fit into these models; for instance, they are expected to train and compete at a considerably greater rate than non-professional athletes (e.g., MLB players play 162 regular season games), and typically have greater access to financial resources and multi-disciplinary support systems.

Missing from available models and frameworks is the recognition that individuals who successfully ascend to the professional ranks of sport continue to develop. Instead, there is an implicit assumption that these athletes have reached the zenith of their capabilities. This notion portrays them as finished products, left to navigate their careers independently. Structured training and programming can (and should) look markedly different when an athlete enters the professional stage of their career. This notion, that once an athlete has reached world-class and professional rankings, their development has been completed, does not align with evidence within and beyond sport. In a non-athlete sample, (Donnellan et al. 2007) noted that significant mental development occurred between the ages 17 and 27, reflecting increased functional maturity during this transition. This has obvious relevance for athletes, emphasizing the need for continued and structured development to meet individuals' needs as they move through these transitions.

A related concept is reflected in the study of “peak ages” for certain sports. For example, (Brander et al. 2014) found that the typical years of peak performance (as measured by points and minutes contribution) in NHL players fell between 24 and 34 years. Similarly, (Allen and Hopkins 2015) examined the typical ages in which different athletes achieved their peak performances (as represented by sport specific outcomes such as world rankings, international competitions etc.), noting a general observation that sports requiring a combination of physical and technical skills, such as ice hockey, tended to have a broader range of peak performance ages. (Baker et al. 2013) noted emerging evidence suggesting high levels of cognitive and motor performance can be maintained longer than previously thought, especially with continued engagement in the activity. For example, (Charness 1981) found that high levels of chess performance could be maintained as performers got older and examinations of other cognitive-motor experts [e.g., handball goalkeepers (Schorer and Baker, 2009) and golfers (Baker et al., 2007)] substantiate these findings. Closely connected is the work by (Baker et al. 2013) who examined career length differences among various positions in different professional North American sports and their performance outcomes. Unsurprisingly, there was a consistent relationship between performance outcomes (e.g., goals per game, assists per game, and penalty minutes per game for ice hockey) and career length, suggesting players with better performance end up with longer careers. Variations in peak performance windows across different sports provide insight into the erraticism within developmental pathways. In sports with shorter performance windows, or those that favor younger athletes, the combination and timing of developmental strategies can differ significantly from sports where peak performance occurs later or spans a longer period. That said, more rigorous and extensive models are required to understand the key characteristics for developmental windows while allowing for nuanced understanding of inter-individual differences. Given how variable human development is (i.e., high degree of inter-individual difference), combined with the variability in the demands of each sport (i.e., high degree of inter-sport variability), there is benefit from establishing more sport-specific development models, as a one-size-fits-all approach likely is not specific enough to guide practitioners' practice and approach.

To help address this, (Battochio et al. 2016) examined the stages and demands of Canadian National Hockey players. In their framework, athletes enter the professional ranks and progress through three distinct stages: Rookie, Unestablished Veteran, and Established Veteran. Each of these phases has distinct physical, psychological, and social challenges, and as a result development strategies should be carefully constructed to meet the individual demands of each athlete at each level. It is expected that an athlete's life circumstances, physical condition, and playing demands will change during his/her/their professional careers. (Gulbin J. et al. 2013), for instance, found that 83.6% of Australian athletes experienced non-linear trajectories. This complexity makes it even more challenging to create models that effectively guide professional athlete development, particularly when existing models fall short of addressing these multifactorial challenges.

Ultimately, the field lacks the theoretical structure and modeling necessary to inform decision-making and guide practice at professional levels. No current models fully address the unique challenges faced by professional athletes or offer sufficient utility to athlete development professionals through an integrative, multi-factorial approach. This absence of theoretical structures that address the unique experiences and needs of NAPS athletes limits opportunities to support athlete development. Developing and structuring specific conceptual models tailored to meet varying demands of professional athletes is crucial for enhancing player development. In particular, models need to recognize the change in factors over time, the intertwined nature of genetics and the environment, and the fact that that a one-size-fits-all approach will not work.

### Challenge 4—implementation science barriers

In many fields, sport included, it takes considerable time for research to become integrated, with much of the research never reaching widespread use (Morris et al., 2011). As a way to combat this lag and foster faster integration from evidence-informed research to application, the field of implementation science (IS) was formed (Wilson et al., 2017). In the context of sport science research, coaches and sport programs need to move as quickly as possible to maintain (perceived) competitive advantages, and often do not have the luxury of time to conduct rigorous evaluations on situations before having to make an important decision. In addition, the translation of important findings may not always be clear enough for interpretation, making it difficult for many interest-holders to appropriately apply the information. This can lead to end users of the information (such as coaches and sports practitioners) left with language-heavy, complex, and abstract understandings of what the research means. This can be seen in the increased in use of computational analysis, data analytics and informatics, particularly within empirical research. This approach provides complex outputs that are hard to translate to end-users and can undermine the relationship between researchers and practitioners, limit access to future research opportunities, and reduce credibility in the eyes of practitioners (Loturco, 2023).

(Loturco 2023, p. 1) highlights some of the typical comments that have been observed from coaches within high performance, which include, “*I avoid interactions with researchers because they often fail to effectively address the issues that impact my daily work*,” “*I don't utilize the scientific services offered because conclusions and recommendations are vague and abstract*,” or “*Results lack clarity and the methods employed are too complex to be implemented at the speed and scale required within (my) professional context*.” Adding to the challenges with research implementation, is the degree of protectionism that organizations often have on the information they collect. Even when research is conducted within NAPS, results are often withheld to maintain a competitive edge, a practice that hampers the scientific pursuit of knowledge. This inhibits an ecosystem of mutualism between the researchers and practitioners, as research often becomes siloed, and valuable work either remains unpublished or lacks the scrutiny and insight provided by the peer-review process. As well, when each team/organization secretly conducts research on athlete development practices, it limits the ability to establish common practices and gold standards.

(Ranaweera et al. 2024) presented a case study that helps illuminate avenues for developing clear guidelines for how professional staff can support athletes. Their case study reviewed existing literature and interviewed performance staff to understand what factors are considered when identifying unusually high (“peaks”) or low (“troughs”) training demands. They then brought practitioners together to agree on a shared set of indicators for interpreting this information. The group ultimately reached consensus on 12 key indicators to help staff more consistently manage player workload in a professional rugby environment. An approach like this (a) helps to improve decision quality by bringing multiple perspectives together reduces the risk of relying on a single metric or opinion and leads to more balanced, informed decisions, (b) creates a shared understanding and continuity for interest-holders reducing confusion, mixed messaging to athletes, and inconsistent workload decisions across departments, and (c) likely increases buy-in and accountability.

(Ranaweera et al. 2025) acknowledged that professional sports teams are always looking for new ways to gain an advantage, often using new technology, research, and data-driven strategies. Staff working in these environments are usually the first to test new ideas and are responsible for turning them into practical tools that improve performance. The authors suggest using a “fail fast, learn fast” approach, meaning teams should quickly test new ideas on a small scale to see if they actually add value before fully investing in them. By creating a simple early version of a new tool or idea and measuring whether it works, teams can decide whether to move forward or adjust their approach. This process helps organizations learn quickly and avoid wasting resources on innovations that do not deliver meaningful benefits.

Improving athlete development should be a common goal with all interest-holders working together to create meaningful and accessible information for change. Encouragingly, a growing trend in professional sports is the hiring or contracting of academic researchers to integrate within NAPS, as well as establishing their own research performance staff/departments to alleviate data collection burdens and positively contribute to high methodological standards. That said, the addition of these new research teams does not necessarily mean there will be more open and shared science, as priority may still be on improving the specific teams' performance rather than advancing the broader research area.

## Discussion

Researchers and practitioners find themselves at a critical juncture for understanding and establishing practices that better support professional athletes in their development. As the resources invested in athlete development increase, and attention on the benefits and risks of participation in elite sport grows, there will almost certainly be increased pressure for professional organizations to systematically engage in “best practices” for developing athletic capability while also addressing the personal, psychological and social developmental needs of their athletes. However, the motivations underlying this increased emphasis on development are not uniform. For some interest-holders, investment in athlete development reflects a genuine commitment to holistic athlete growth and long-term wellbeing. For others, such investment may be primarily driven by performance optimization, asset protection, and financial return within a highly commercialized sport system. In professional sport, athletes represent both developing individuals and organizational assets, creating a potential tension between developmental support and performance imperatives. These competing priorities shape the context within which frontline practitioners operate, often requiring them to navigate complex organizational expectations while attempting to advocate for athlete-centered approaches. Recognizing this structural tension is critical for understanding both the opportunities and constraints surrounding professional athlete development.

In this narrative review, four significant challenges facing athlete development within NAPS were identified. Table 1 builds on the identified challenges, and suggests ways to better support professional athlete development within these constraints.

**Table 1:** Overview of challenges and potential approaches for overcoming those challenges.

Challenge	Potential solutions and approaches
Definitional ambiguity	- Ensure organizational alignment in policy and practice documents, so all interest-holders are clear on what development means and how it can be measured. Reaching a consensus on important terms is critical for developing a systematic operational framework that can guide both research and practice - Those working within NAPS should strive to secure buy-in from top leadership. Clear commitment from senior management is critical for any the success in determining alignment and implementation efforts - Discourage the use of terminology that is frequently interchanged with other terms, or that has vague definitions. For instance, talent development could be changed to athlete development for clarity
Measurement challenges	- Designate individuals responsible for managing data within athlete development departments - Use methodologies and statistical applications that consider the complexities of professional sports. Consider a multifaceted approach to evaluating and measuring development. Athlete development is inherently multifaceted, involving social, emotional, psychological, motor/skill, cognitive/tactical, and physical factors; measurement for development should consider these factors if it is to be representative and accurate - Embrace small sample analyses. Those studying elite athletes should recognize that recruiting the necessary sample size for commonly accepted analysis methods may be unlikely, and thus they should invest in developing knowledge in methodologies and procedures designed for small sample sizes - High-quality research often takes time, whereas rushed research and poor results erode confidence and undermines academic integrity. Patience is needed to produce accurate insights that may are reproducible
Restrictive theoretical modeling	- Create athlete development models reflecting the specific needs of professional athletes. This sub-set of the population should be treated as “works in progress.” There is a need for more sophisticated and adaptable models that consider the continuous development required at professional levels, acknowledging that athlete growth is a lifelong process influenced by various factors - Consider the voices and lived experiences of practitioners and athletes for modeling development at the professional level
Implementation science barriers	- Embed professionals with implementation expertise who can liaise between researchers and practitioners; this will help ensure that research findings are translated into practical application. Individuals who would appropriately fit a role such as this would have a deep understanding of both scientific practices as well as the operational realities of NAPS environments - Access to academic journals is often difficult for practitioners, who may lack the time or expertise to navigate the array of publications available. Researchers should strive to make their findings accessible and user-friendly, advocating for open access to eliminate barriers to knowledge consumption - Promote collaborative research projects. Foster systems where research projects are developed and executed involving both academics and practitioners from the outset. This may help develop meaningful research questions which are relevant to practical needs - Develop standardized implementation processes. To create a sustainable and impactful environment, NAPS organizations should collaborate with researchers to develop flexible yet structured protocols for implementing new research findings and interventions - Ensure research outputs have a section or multiple sections on “how this information can be used for practitioners” to encourage practical, user-friendly insights

## Limitations

While there is value in exploring athlete development from a lifespan perspective (i.e., including the period after peak performance and retirement), the present review was exclusively concerned with the development of athletes who have already achieved elite levels within NAPS. Although there may have been some limitations to the search strategy used (e.g., using a narrative approach instead of a systematic or scoping review approach), the nature of the research question combined with the limited resources available for professional level athlete development required a broad investigation through an exploratory approach.

Another limitation was that the authorship was English-speaking, and as a result, the nature of the articles and references collected were from English written resources only. There may have been other articles that would fit the criteria in other languages that were missed. Similarly, given professional women's sport is relatively new in the North American systems, articles focused on women's development were limited. It would be valuable to revisit this research question in five to 10 years, as research is likely to grow considerably as the number of teams and programs continue to expand.

A further limitation of the present review relates to the inconsistency of terminology used within the field. As discussed earlier, terms such as professional, elite, expert, high-performance, and world-class are often used interchangeably despite reflecting distinct constructs (e.g., employment status, performance standard, or expertise attainment). This lack of terminological precision created challenges in developing a comprehensive search strategy. Although efforts were made to include a broad range of relevant terms, it is possible that studies meeting the intended criteria were not captured due to inconsistent labeling within titles, abstracts, or keywords. The conflation of these constructs not only risks obscuring theoretically meaningful distinctions but also contributes to the fragmented nature of research in this area. Greater conceptual clarity and alignment among researchers and practitioners would enhance the coherence, comparability, and cumulative advancement of knowledge in professional athlete development.

## Conclusion

The example of Leo Carlsson and the Anaheim Ducks highlights the increasing importance professional organizations place on player development, underscoring a significant opportunity for expanded research into this previously underexplored area. As research into elite athlete development continues to grow, it requires careful methodological attention. This review discussed the complexities involved in studying elite athletes, such as sample size challenges, the need for longitudinal study designs, and the critical role of robust measurement techniques. By acknowledging and addressing these challenges, the field of athlete development within NAPS may take another step forward in finding athlete-centered strategies to encourage growth and career longevity in athletes. Collaboration between researchers and practitioners, along with a commitment to clarity and precision in terminology and measurement, will help ensure that we can better support the athletes under our care. This collective effort will help build meaningful insights and develop practical strategies that support the growth and success of athletes at all levels of competition.
